# Effectiveness of pharmacologic therapies on smoking cessation success: three years results of a smoking cessation clinic

**DOI:** 10.1186/2049-6958-9-9

**Published:** 2014-02-04

**Authors:** Elif Yilmazel Ucar, Omer Araz, Nafiye Yilmaz, Metin Akgun, Mehmet Meral, Hasan Kaynar, Leyla Saglam

**Affiliations:** 1 Department of Pulmonary Diseases, Ataturk University School of Medicine, Erzurum, Turkey; 2 Yakutiye Medical Research Center, Chest Disease Department, Erzurum, Yakutiye 25240, Turkey

**Keywords:** Effectiveness, Pharmacologic therapy, Quit rate, Smoking cessation

## Abstract

**Background:**

Pharmacologic therapies have an important role in the success of interventions for smoking cessation. This study aims to determine the efficacy of several pharmacologic treatments in patients who applied to a smoking cessation clinic.

**Methods:**

This retrospective study includes 422 patients who presented to our smoking cessation clinic between January 2010 and June 2013, used the pharmacologic treatment as prescribed and completed the one-year follow-up period. All patients were assessed using the Fagerström Test for Nicotine Dependence (FTND) and received both behavioral therapy and pharmacotherapy. Patients’ smoking status at one year was assessed by telephone interview.

**Results:**

The patients were 24.3% female (103/422) and 75.7% male (319/422) with a mean age of 38 ± 10 years. Patients were divided into three groups: varenicline (166 patients), bupropion (148 patients) and nicotine replacement therapy (108 patients).

The smoking cessation rates of these groups were 32.5%, 23% and 52.8%, respectively, and were statistically significant (p > 0.001). The overall success rate was 35%. Further analysis revealed that pharmacologic therapy (p > 0.001) and gender (p = 0.01) were factors that showed statistically significant effects on smoking cessation rates. Males had higher success rates than females. The overall relapse rate was 21.6% and the bupropion group showed the highest relapse rate among treatment groups. Lack of determination emerged as the most important factor leading to relapse.

**Conclusion:**

Nicotine replacement therapy was found to be more effective at promoting abstinence from smoking than other pharmacologic therapies.

## Background

Smoking is the leading cause of preventable morbidity and mortality worldwide [1,2]. Smoking cessation significantly improves life expectancy, decreases morbidity, and reduces healthcare costs associated with smoking-related conditions [3]. Interventions that include both counseling and pharmacotherapy appear to be the most effective, and success is more likely with more intensive interventions [4,5].

There are several pharmacological interventions available to aid smoking cessation [6]. The Food and Drug Administration (FDA) has approved seven medications for this purpose: five nicotine replacement therapies (NRT), bupropion and varenicline. In addition, the antihypertensive medication clonidine and the tricyclic antidepressant nortriptyline are sometimes used as second-line agents for smoking cessation, but their use is not FDA approved for this indication [7]. The most widely used product in Europe and the United States is NRT, which increases smoking cessation rates at one year by approximately 70% [6,8]. Clinical trials have demonstrated statistically significant improvement in smoking cessation with varenicline and bupropion [9,10].

The aim of this study was to evaluate the efficacy of NRT, varenicline and bupropion for smoking cessation.

## Methods

### Study population

Four hundred and twenty-two active smokers who presented to Ataturk University smoking cessation clinic between January 2010 and June 2013, received pharmacological therapy, and completed one year follow up, were included in the study. Patients who did not attend follow up visits, did not use their medication regularly as prescribed or were not available for a phone interview at the end of the one-year follow up were excluded from the study.

### Study design

In this retrospective study, data were collected by reviewing medical records in an existing clinical database. All demographic, clinical and laboratory findings, results of the Fagerström Test for Nicotine Dependence (FTND)**,** as well as treatment choices and their outcomes**,** were recorded. Patients were categorized into three treatment groups: varenicline, sustained-release bupropion and NRT, which included nicotine patch and nicotine gum. In addition to one of these supporting drugs, all patients underwent behavioral therapy. The local ethics committee of Ataturk University, Faculty of Medicine approved the protocol of the study.

### Pharmacological regimen

In the NRT group, the 24-hour nicotine patch delivery system was used to aid smoking cessation. Subjects who smoked more than 20 cigarettes per day (CPD) began with a 21 mg/d nicotine patch for the first two weeks, 14 mg/d in weeks three to six and 7 mg/d in weeks seven to twelve. Nicotine gum was used when needed to fight cravings. In the varenicline group, patients received 0.5 mg/d varenicline for three days, followed by 1 mg/d (0.5 mg twice daily) for four days. On day 8, the target quit date, the varenicline dose was increased to 1 mg twice daily. The duration of treatment was a total of 12 weeks. In the bupropion group, patients received sustained-release bupropion at 150 mg/d for three days, then 150 mg twice daily for the remaining 12-weeks.

### Definition of relapse

To resume smoking after 3 months or more of smoking cessation.

### Statistical analysis

Statistical analysis was performed with SPSS for Windows version 17.0 (SPSS Inc., Chicago, USA). Data were expressed as percentage, mean and standard deviation, odds ratio and 95% confidence interval. We used Pearson Chi square test for comparisons of nominal variables and ANOVA for numeric variables. We performed an analysis adjustment for confounders using logistic regression. Findings were considered statistically significant at p > 0.05.

## Results

**Figure 1:**
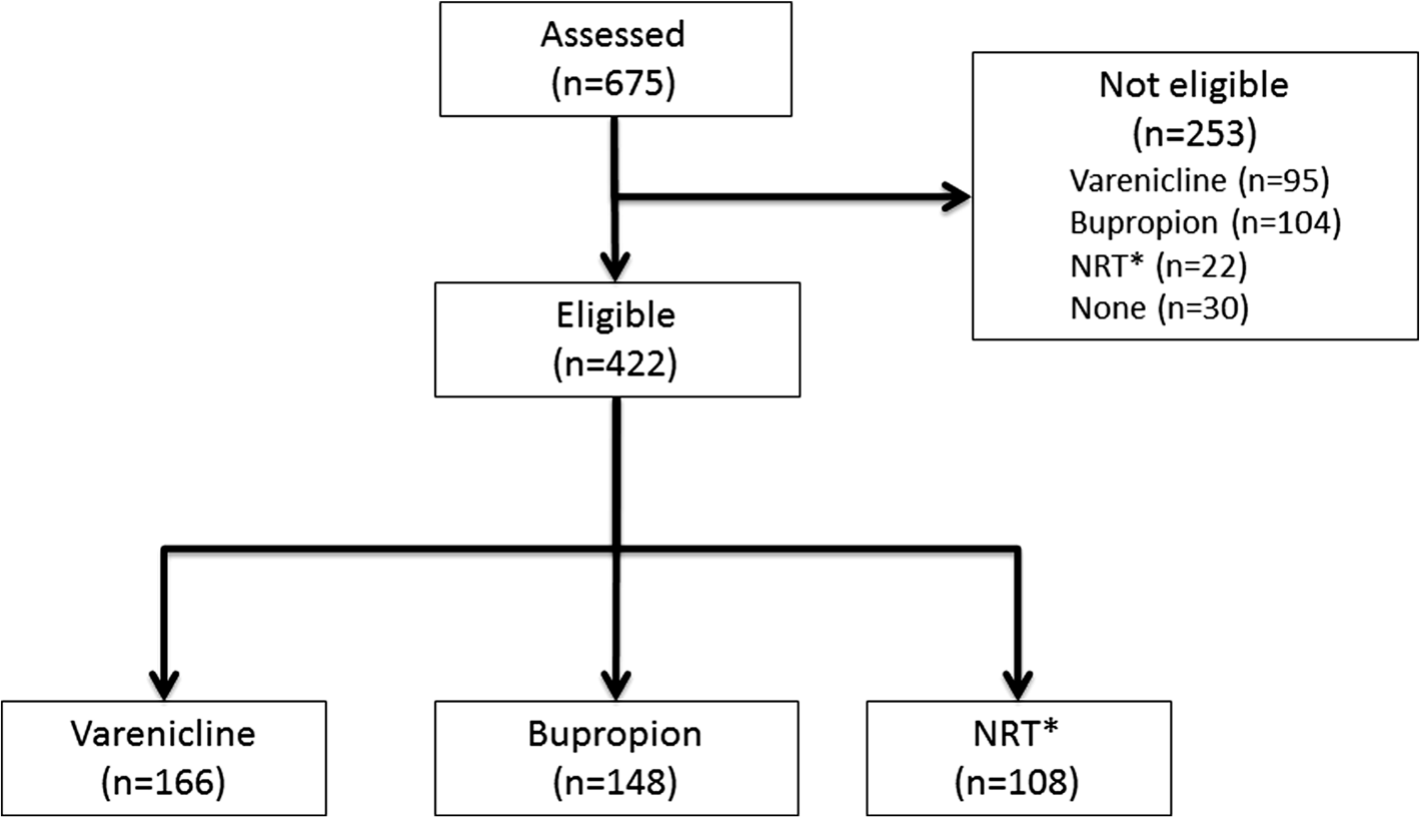
**Study diagram.** *NRT, Nicotine replacement therapy.

From January 2010 to June 2013, a total of 675 patients were assessed and 422 out of them, 103 (24.3%) females and 319 (75.7%) males (mean age: 38 ± 10 SD) with active smoking were included in the study. Out of these, 166 (38%) received varenicline, 148 (35%) bupropion and 108 (27%) NRT (Figure 1). There was a significant difference between groups in smoking habit at time of admission to the clinic. Smoking pack/day, pack/years and FTND were highest in the varenicline group (p > 0.001). The patients’ characteristics are shown in Table 1.

**Table 1:** Patients’ characteristics at admission

	**Treatments**			
Parameters	Varenicline (n = 166)	Bupropion (n = 148)	NRT (n = 108)	p
Age, years (mean ± SD)	39 ± 9	36 ± 11	38 ± 12	0.86
Female/Male	36/130	37/111	30/78	0.50
Smoking start age, years	16 ± 4	16 ± 5	16 ± 5	0.92
Cigarettes pack/day, mean ±SD	1.4 ± 0.56	1.2 ± 0.47	1.1 ± 0.53	0.003
Smoking pack/years	30.3 ± 20.9	22.9 ± 15.9	25.1 ± 18.6	0.001
mean ± SD				
Smoking start reasons				
Imitating/curiosity	70/57	74/43	38/45	0.02
Marital status (Single/Married)	21/145	36/112	19/89	0.02
Patient request to quit smoking	158	146	103	0.08
Educational status	45	33	37	0.04
Primary school	55	49	37	
High school	66	66	34	
University				
FTND score, mean ± SD	7.1 ± 2.4	6.4 ± 2.4	5.4 ± 2.6	0.001

FTND; Fagerström test for nicotine dependence.

The overall smoking cessation rate after 1 year was 35%. The smoking cessation rates for varenicline, bupropion and NRT were 32.5%, 23%, and 52.8%, respectively, and were statistically significant (p > 0.001) (Table 2).

**Table 2:** Rates of quit smoking and relapse according to treatments at the end of one year

**Treatments**	**Quit smoking**		**Relapse**	
	**N**	**%**	**N**	**%**
**Varenicline**	**54**	**32.5**	**38**	**22.9**
Bupropion	34	23	47	32
**NRT**	**57**	**52.8**	**6**	**5.6**

NRT, Nicotine replacement treatment.

Due to differences among treatment groups in patient’s smoking habits at the time of admission, logistic regression analysis was conducted with factors affecting smoking cessation success rates. Age, marital status, education level, age at smoking onset, reason for beginning to smoke, history of cessation, smoking pack/years, cigarette pack/day, and FTND score did not have a positive or negative effect (p < 0.05). Being male had a positive effect on success (p = 0.01); treatment type also had a significant effect on success (p > 0.001) (Table 3).

**Table 3:** Factors affecting success in quit smoking

**Factors**	**p**
Age	0.99
Smoking pack/years	0.20
Smoking pack/day	0.41
FTND score	0.11
Sex	0.01
Treatments	> 0.001

FTND; Fagerström test for nicotine dependence.

The overall relapse rate was 21.6%. The relapse rates for varenicline, bupropion and NRT groups were 22.9%, 32%, 5.6%, respectively and were statistically significant (p > 0.001) (Table 2). The most frequent factors leading to relapse were lack of determination (29.4%), social pressure (22.9%) and stress (9%).

## Discussion

This study showed that, besides behavioral therapy, pharmacologic treatment is important in smoking cessation success rates. It was found that NRT is the most efficient pharmacotherapy agent for smoking cessation. Relapses remained a major problem in the smoking cessation process. The bupropion group showed the highest relapse rate compared to varenicline and NRT groups, and lack of determination was the most important factor associated with relapse.

Smoking cessation is physically and psychologically challenging. Most smokers desire to quit, but without assistance the success rate is very low (4-7%) [4,5]. Intervention options to aid smokers in cessation are few. Interventions which include both behavioral and pharmacological therapy have been shown to be most effective, with success rates between 22 and 45% [11-15]. In the current study, the success rate was 35%.

Recently a meta-analysis demonstrated that varenicline, bupropion and NRT were all more effective than placebo in promoting smoking abstinence at one year [8,16]. Varenicline, the most recent pharmacologic therapy for smoking cessation, has been available since 2006. It exerts its effect as a nicotinic receptor antagonist. In two wide ranging, multicenter, double blind, phase III, randomized clinical studies, varenicline was shown to be safe and effective in promoting both continuous abstinence and long-term cessation [9,17]. Gonzales et al. compared varenicline and placebo at the end of three months and found that cessation rates were 44% and 18%, respectively (OR:3.85, 95% CI: 2.70-5.50) [9]. In the same study, the comparison of success rates between bupropion and varenicline groups was not statistically different, although varenicline was found to be more effective than the other drug (21.9% vs 16.1%, OR: 1.46, 95% CI: 0.99-2.17). In studies comparing varenicline to NRTs, varenicline was found to be significantly more effective than a standard dose of NRT (56% vs 43%, respectively; OR: 1.70) [18,19]. However, in studies using combined NRTs (such as nicotine patch plus nicotine gum or nasal spray), varenicline and NRT showed comparable results (NRT: 37%; varenicline: 33%) [4]. In the current study, NRT was significantly more effective than varenicline based on patients’ smoking cessation rates at one year (32.5% vs. 52.8%). This result suggests that NRTs may be a cost-effective first-line treatment.

Buproprion SR was the first nicotine-free drug that was proven to help fight nicotine addiction. It exerts its effect by blocking the release of dopamine and noradrenaline and inhibiting nicotinic acetylcholine receptors [20]. In randomized-control studies bupropion was found to be twice as effective as placebo in aiding smoking cessation [20]. In another study, at 6 months the smoking cessation rate of the bupropion group was higher (27%) than that (16%) of the placebo group [21]. When bupropion treatment was combined with behavioral therapy, the smoking cessation rate at one year was found to be twice that of the placebo group [22].

Clinical trials have demonstrated significantly higher rates of smoking cessation for varenicline than for bupropion [9,10,17], though a recent meta-analysis has demonstrated the superiority of bupropion to standard doses of NRT in promoting cessation at both three months and one year [16]. However, when bupropion was compared to NRTs, there was no significant difference in cessation rates at one year. In the current study, NRT was found to be more effective than bupropion (52.8% vs. 23%). The combination of NRT and behavioral therapy may explain the increase in success rates. The fact that the success rates of all treatment groups at one year were higher in the current study than those reported in the literature may be attributable to the concurrent use of behavioral therapy.

Relapse is a major problem for people attempting to quit smoking. It has been observed that pharmacotherapy has a limited effect on preventing patients from resuming smoking. Treatment with NRTs has been shown to more effectively aid initial abstinence compared to bupropion [23]. In studies that have attempted to prevent relapse by prolonging varenicline and bupropion treatment, only varenicline showed a preventative effect [24]. In the current study, the highest rate of relapse was in the bupropion group (32%). The main factors leading to relapse were the patients’ lack of determination and social pressure. This suggests that the ability of pharmacotherapy to prevent relapse is limited.

Although the retrospective study design has some limitations, analyzing the clinical outcomes of real-life treatment options provides valuable information for future therapeutic practices.

## Conclusions

In conclusion, NRTs can be used as the first treatment choice in the absence of contraindications. Factors leading to relapses require further assessment, and tailored behavioral therapy might be considered to deal with relapses.

## Competing interest

The authors declare that they have no competing interests.

## Authors’ contributions

EYU, Assistant Professor Doctor, data analysis, design of the study. OA, Assistant Professor Doctor, acquisition of data. NY, Specialist, acquisition of data. MA, Professor Doctor, edit manuscript. MM, Professor Doctor, acquisition of data. HK, Professor Doctor, acquisition of data. LS, Professor Doctor, edit manuscript. All authors read and approved the final manuscript.
